# Detection and quantification of drugs on banknotes by LC–MS/MS with a fast and non-destructive sample preparation: a comparison of three cities

**DOI:** 10.1007/s11419-025-00711-w

**Published:** 2025-01-30

**Authors:** Göksun Demirel, Yeter Erol Öztürk, Oya Yeter, Hızır Aslıyüksek

**Affiliations:** 1 Department of Pharmaceutical Toxicology, Faculty of Pharmacy, Cukurova University, Adana, Turkey; 2 Department of Forensic Sciences, Institute of Addiction and Forensic Sciences, Cukurova University, Adana, Turkey; 3 Chemistry Department, Council of Forensic Medicine, Ankara, Turkey; 4 Chemistry Department, Council of Forensic Medicine, Istanbul, Turkey; 5 Morgue Department, Council of Forensic Medicine, Istanbul, Turkey

**Keywords:** Drug residues, LC–MS/MS, Contamination, Paper currency, Drug Abuse

## Abstract

**Purpose:**

The analysis of drug residues on some currencies is well-established in the literature. However, there is no published study describing the presence of drug residues on Turkish paper currency.

**Methods:**

This study focused on the analysis of 14 drug residues present on 600 Turkish banknotes collected from three different cities: Ankara, Adana, and Istanbul. The banknotes underwent preparation by a non-destructive and straightforward extraction method using methanol. To investigate the extent of contamination a method was subsequently developed and validated for liquid chromatography triple quadrupole mass spectrometry analysis to detect and quantify the target analytes. The investigated substances included benzoylecgonine, cocaine, heroin, codeine, morphine, 6-monoacetylmorphine (6-AM), amphetamine, methamphetamine, 3,4-methylenedioxy-N-methamphetamine (MDMA), methyl 3,3-dimethyl-2-(1-(pent-4-en-1-yl)-1H-indazole-3-carboxamido)butanoate (MDMB-4EN-PINACA), N-[1-(aminocarbonyl)-2,2-dimethylpropyl]-1-butyl-1H-indazole-3-carboxamide (ADB-BUTINACA), tetrahydrocannabinol (THC), pregabalin, ketamine, and tramadol.

**Results:**

The calculated mean concentrations per note were 475.5 ng cocaine, 660.7 ng methamphetamine, 220.4 ng benzoylecgonine, 36.5 ng ketamine, 46.0 ng amphetamine, 120.6 ng 6-AM, 22.9 ng morphine, 6.3 ng codeine, 107.4 ng THC, 1.3 ng MDMB-4en-PINACA, 1.1 ng ADB-BUTINACA and 65.9 ng MDMA. Our findings indicate that banknotes commonly circulated in the three cities were primarily contaminated with methamphetamine and cocaine.

**Conclusions:**

This study highlights the prevalence of drug residues on banknotes and raises concerns about their potential impact. The contamination of Turkish currency with drug residues is a strong indication of the widespread use of banknotes in drug trafficking.

## Introduction

Global surveys have revealed a continuous rise in the abuse of both illicit and licit drugs. The 2019 report from the United Nations Office on Drugs and Crime (UNODC) indicated that approximately 271 million people used illicit drugs in 2017, reflecting a 30% increase since 2009 [1]. A subsequent UNODC report in 2023 indicated that approximately 296 million individuals, aged 15–64 years, used drugs globally in 2021 [2]. The pervasive distribution of illicit drugs worldwide has become a serious problem with profound implications for public health, crime rates, and road safety [3].

Recent studies indicate that banknotes contaminated with illicit drugs are also circulating worldwide [4–6]. Drug contamination is believed to originate from drug abuse [7] and can spread through direct contact by hand or via contaminated currency-counting machines and automated teller machines (ATMs) in banks [8]. The presence of illicit and licit drugs in circulating currency poses a potential source of exposure to the public through skin contact and inhalation, especially among employees working with currency sorting machines [9]. Banknotes also serve as a valuable tool for forensic epidemiology, as the detection of drugs in currency provides information on trends or changes in drug use [10]. Analyzing drug traces in banknotes may reveal the types of drugs used in a specific area [11]. To identify and strengthen timely plans for safe and appropriate control measures to prevent the spread of illicit drugs in the environment and protect human and ecological health, information on the global prevalence of illicit drugs is essential.

In this study, we aimed to evaluate the presence of drug residues and their concentration ranges on 600 Turkish Lira (TL) banknotes collected from the metropolitan cities of Istanbul, Ankara, and Adana, using a fully validated triple quadrupole mass spectrometry (LC–MS/MS) method. MDMB-4en-PINACA and ADB-BUTINACA were selected for the study because these substances are the most commonly detected synthetic drugs in seizures of narcotic drugs and the analysis of biological samples by the Council of Forensic Medicine. Ketamine, pregabalin, and tramadol are also the most commonly detected licit drugs in seizures of narcotic drugs and the analysis of biological samples by the Council of Forensic Medicine.

## Experimental Design

### Chemicals and Reagents

Benzoylecgonine, cocaine, heroin, codeine, morphine, 6-AM, amphetamine, methamphetamine, MDMA, MDMB-4en-PINACA, ADB-BUTINACA, THC, pregabalin, ketamine, and tramadol were procured from Chiron (Trondheim, Norway). The analytes from Chiron have already been dissolved in methanol with the adjusted final concentration of 1 mg/mL as freebase. The deuterated internal standard (IS), diazepam-d5, was acquired from Cerilliant (Paloma, TX, USA) at concentrations of 1 mg/mL. All reagents and chemicals, including ammonium acetate, formic acid, and methanol, were of LC–MS grade and sourced from VWR Chemicals (Gibbstown, NJ, USA).

### LC–MS/MS Conditions

The chromatographic setup consisted of a Shimadzu 20 C UPLC liquid chromatograph with an integrated column oven (Shimadzu, Kyoto, Japan) coupled to a Shimadzu 8050 triple quadrupole mass spectrometer. Separations were conducted using an Agilent Poroshell (150 × 4.6 mm, 2.7 μm) column maintained at 40 °C. The mobile phase comprised A (water containing 5 mM ammonium acetate and 0.1% v/v formic acid) and B (methanol) at a flow rate of 0.6 mL/min. The gradient, with a total run time of 15 min, was as follows: 0–0.3 min at 10% B; 0.3–3 min from 10 to 80% B; 3–7 min from 80 to 95% B; 7–11 min at 95% B; 11–11.1 min from 95 to 10% B; and 11.1–15 min at 10% B. The injection volume was 5 μL, and the autosampler temperature was 8 °C. The equipment operated in ESI + and multiple reaction monitoring (MRM) modes, with specified parameters: source voltage at 1.5 kV, nebulizing gas flow rate at 3 L/min, heating gas flow at 10 L/min, interface temperature at 300 °C, desolvation line temperature at 250 °C, heat block temperature at 400 °C, drying gas flow at 10 L/min, desolvation temperature at 526 °C, and CID gas at 270 kPa. The dwell time for analytes ranged between 5 and 13 ms, with pause and polarity-switching times set to 1 and 5 ms, respectively. Two different fragment ions were selected with respective intensities.

### Turkish Banknotes

Non-circulating freshly printed banknotes were sourced from the National Bank of Ankara for validation and calibration. A total of 600 banknotes in circulation were collected from Adana, Ankara, and Istanbul. Banknotes of denominations 20, 50, 100, and 200 TL were introduced into circulation in 2009, with 5 and 10 TL banknotes being circulated from 2013 onwards until August 2022. Samples, including 40 each of 5, 10, 20, and 50 TL banknotes, and 20 each of 100 and 200 TL banknotes, were randomly collected from bus stations, open bazaars, train stations, grocery stores, and ATMs in three cities over 20 days in August 2022. After collection, banknotes from each city were bundled (10–20 notes), handled with nitrile gloves, and packed in plastic bags. Statistical analysis of mean differences within cities and contamination levels of drugs in different denominations (5, 10, 20, 50, 100, and 200 TL banknotes) were evaluated using one-way ANOVA, considering a *p*-value < 0.05 as significant.

### Extraction Procedure

Banknotes were placed in polypropylene tubes with 10 mL of methanol, and 50 µL IS (20 ng/mL) was added to each tube. The tubes were sealed, shaken at 200 rpm for 10 min, and sonicated for an additional 10 min. After removing the banknotes, the extracts were evaporated, ensuring no damage to the security band or holographic marks. After evaporation at 40 °C under a stream of nitrogen, residues were reconstituted in 500 µL water: methanol (80:20 v/v), followed by vortexing for 2 min. The extracted solids were removed by centrifugation (10 min, 14,000 rpm). The supernatant was transferred to vials and analyzed by LC–MS/MS.

### Method Validation

Freshly printed, non-circulating 5 TL banknotes were used to validate this method. A methanolic analyte mixture with a concentration of 1 mg/L was prepared and stored at – 20 °C. An eight-point calibration in the 0.5–100 ng/note range for all analytes (0.5, 1, 5, 10, 25, 50, 75, and 100 ng/note) was applied after spiking and drying banknotes for 2–4 h. Linear regression with 1/x weighting was employed. LOD (signal to noise ≥ 3) and LOQ (signal to noise ≥ 10) were estimated as the lowest concentrations with acceptable precisions from spiked samples (n = 6). Repeatability was determined by performing six replicates (50 ng/note) on the same day, and reproducibility was assessed by performing six replicates for 5 consecutive days (50 ng/note). Matrix effect (ME), recovery (RE), and process efficiency (PE) were calculated following the methods described by Matuszewski and Constanzer with six consecutive injections [12].

## Results

### Method Validation Results

Good linearity was consistently observed with an R^2^ value exceeding 0.99 in all cases. The calculated precisions were acceptable, with repeatability precision ranging from 1.2% to 8.8% RSD, and reproducibility precision ranging from 2.3 to 10.1% RSD. The LODs ranged from 0.3 to 4.1 ng/note, and the LOQs were in the range of 0.5 to 5.3 ng/note. Extraction recoveries ranged from 61.8 to 97.6%. MEs and PEs exhibited values within the range of 67.3–146.1% and 73.6–159.2%, respectively. Detailed validation results are presented in Table 1.

**Table 1:** Validation results

Compound	Linearity (R^2^)	Repeatability (RSD%)	Reproduciblity (RSD %)	LOD (ng/note)	LOQ (ng/note)	Recovery (%)	Precursor/Quantifier/Qualifier Ions
ADB-BUTINACA	0.9989	1.9	2.3	0.3	0.5	91.4	331.2/145.0/201.0
MDMB-4en-PINACA	0.9998	1.8	2.5	0.3	0.5	81.8	358.1/213.0/298.0
THC	0.9986	8.8	10.1	1.8	2.6	74.2	315.2/193.0/259.0
Amphetamine	0.9971	4.5	5.3	0.9	1.4	72.3	136.1/91.0/119.0
Methamphetamine	0.9915	2.8	4.2	0.9	1.4	65.5	150.1/91.0/119.0
MDMA	0.9967	1.2	3.4	0.7	1.0	71.1	194.1/163.0/105.0
Cocaine	0.9976	3.0	3.2	0.4	0.6	70.4	304.1/182.0/82.0
Benzoylecgonine	0.9973	2.2	4.3	0.8	1.2	67.1	290.1/168.0/82.0
Ketamine	0.9989	1.9	2.6	0.6	0.8	71.0	238.1/125.0/179.0
Tramadol	0.9954	1.7	2.5	0.5	0.7	70.0	264.1/58.0/42.0
6-MAM	0.9973	2.8	3.1	0.8	1.1	65.3	328.1/165.0/211.0
Morphine	0.9995	3.3	3.8	0.9	1.4	62.5	286.1/152.0/201.0
Codeine	0.9998	4.6	4.5	1.1	1.5	61.8	300.1/152.0/215.0
Pregabalin	0.9993	3.8	4.3	4.1	5.3	97.6	160.1/58.0/83.0
Heroin	0.9936	2.3	2.9	0.8	1.3	89.6	370.1/165.0/268.0

Bones et al., Wimmer and Schneider, and Bowdler et al. documented instances of illicit and abused pharmaceutical contamination on banknotes using a validated LC–MS/MS method (12, 14, 21). Specifically, Bones et al. reported LOQs that ranged from 0.01 to 3.4 ng/note for morphine, heroin, amphetamine, MDMA, benzoylecgonine, ketamine, cocaine, methamphetamine, and THC on Irish euro banknotes [13]. Wimmer and Schneider reported LOQs between 0.02 and 9.5 ng/note for benzoylecgonine, cocaine, MDMA, MDA, MDEA, methamphetamine, heroin, 6-AM, morphine, and THC on euro banknotes [12]. Bowdler et al. consistently reported all LOQs as 1 ng/note for methamphetamine, MDMA, ketamine, benzoylecgonine, and cocaine on Bristol local euro banknotes. The LOQs identified in our study align with those of previous research [6].

### Turkish Banknote Analysis

**Fig. 1:**
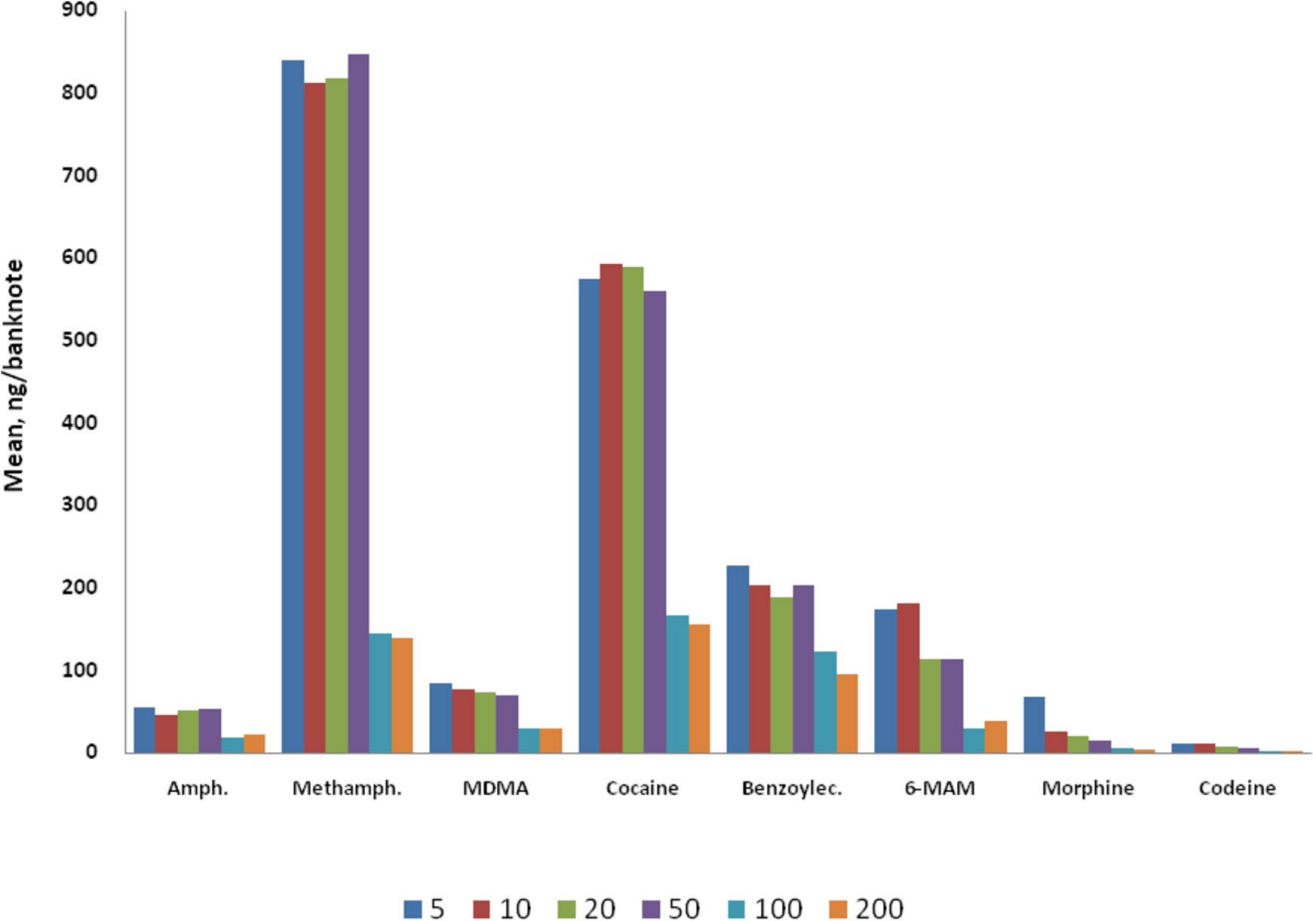
Mean distribution of amphetamine, methamphetamine, MDMA, cocaine, benzoylecgonine, 6-AM, morphine, and codeine with relation to TL denomination

The LC–MS/MS method was applied to detect drug contamination on Turkish banknotes, revealing drug contamination across all analyzed banknotes. The study results indicate widespread contamination of different drugs on banknotes in daily circulation. The typology of drugs varied based on the country, with contamination levels significantly differing between banknotes based on denomination. Amphetamine, MDMA, morphine, and codeine were found in low concentrations on all notes. No trend was observed for benzoylecgonine according to statistical analysis. (Fig. 1).

**Fig. 2:**
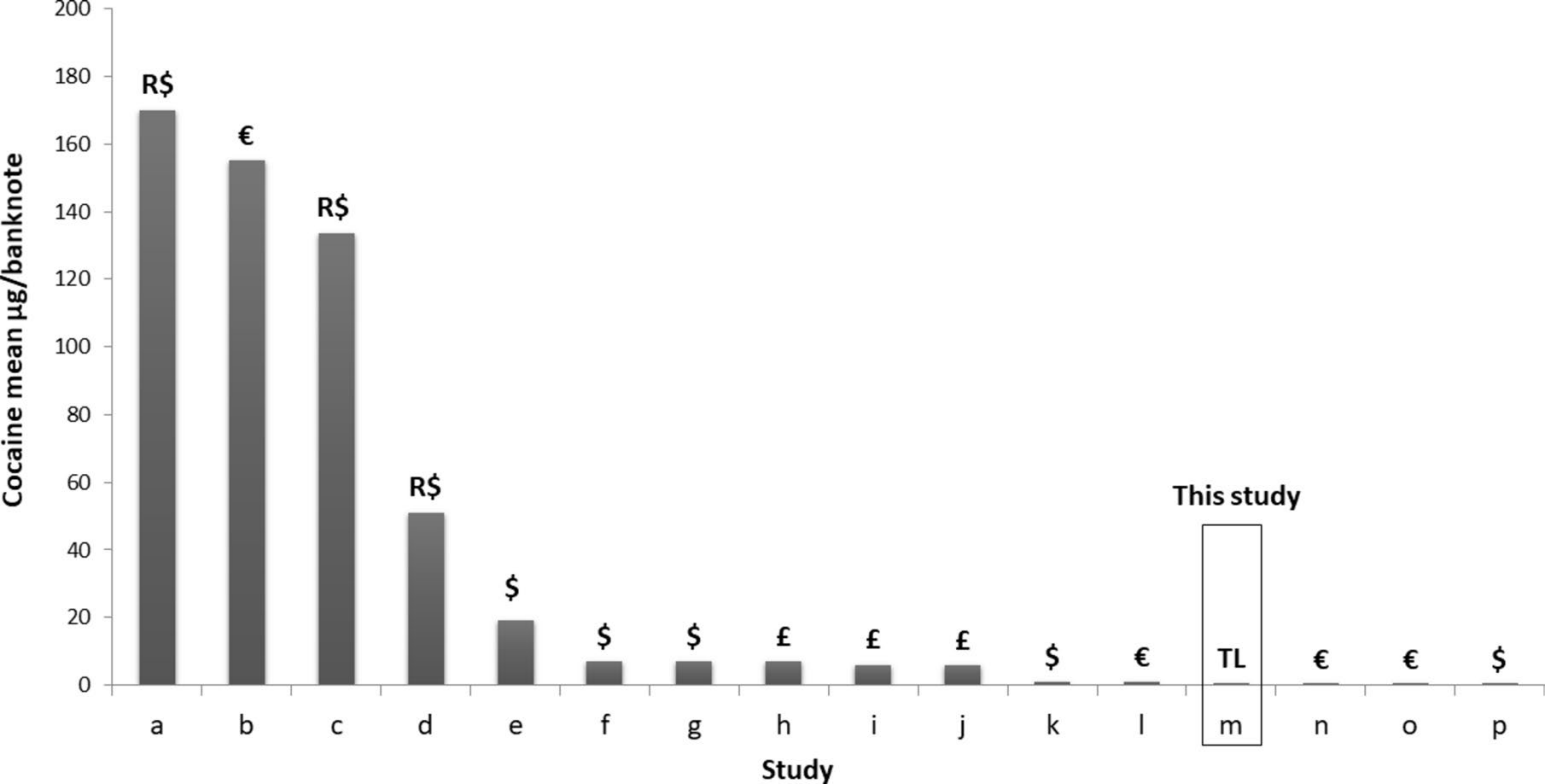
Mean concentrations of cocaine (µg/banknote) reported in various studies: **a** (19); **b** (20); **c** (21); **d** (22); **e** (23); **f** (6); **g** (24); **h** (18); **i** (25); **j** (26); **k** (27); **l** (16); **m** the present study, 2023; **n** (4); **o** (13); **p** (28)

In previous studies, cocaine contamination in banknotes was reported for all analyzed banknotes [5, 6, 11]. The quantity of cocaine per banknote exhibited variability, with average contamination levels ranging from 0.102 to 534 µg/banknote and high levels reaching 1110 µg [14] and 1330 µg [15]. In the present study, cocaine was likewise detected in all banknote samples, with a detection range of 2.3–8438 ng/note and an average concentration of 475.5 ng/note, as illustrated in Fig. 2. Compared to previous studies, the level of cocaine contamination in this study was notably low, and the average cocaine concentration closely resembled that reported by Wimmer and Schneider [4] and Mackul'ak et al. [16]. Ieong et al. reported an average cocaine concentration of 77.9 ng/note [17]. Substantial variation in cocaine concentrations on individual banknotes, ranging from nanograms to micrograms. Various mechanisms have been proposed to elucidate the presence of cocaine on banknotes, including direct contact with the drug during handling, the use of rolled-up notes for snorting, and indirect transfer of drugs from one contaminated banknote to another during counting or storage in the same wallet [18].

The banknotes also showed noteworthy amounts of benzoylecgonine, consistent with findings from previous studies [4, 6, 13, 16–29]. Benzoylecgonine was detected in all banknote samples, with a detection range of 1.0–3241.0 ng/note and a mean concentration of 220.4 ng/note. Ieong et al. and Wimmer and Schneider reported mean concentrations of 34.3 and 43.0 ng/note, respectively, for benzoylecgonine [4, 17]. Originating as the primary degradation product of cocaine, benzoylecgonine is chemically formed through the hydrolysis of cocaine under humid conditions or in the presence of water. Additionally, benzoylecgonine can be derived from contact with sweat or other bodily excretions, such as urine [6, 13, 29].

Ketamine was detected in all banknotes, with a detection range of 0.6–622.4 ng/note and a mean concentration of 36.5 ng/note in the recent study. Bowdler et al. reported a mean concentration of 9.95 µg/banknote for ketamine, while Mackul'ak et al. detected a concentration of < 0.3 ng/note [6, 16]. Ketamine was identified as an adulterant in bulk cocaine samples by the Council of Forensic Medicine. Additionally, Ieong et al. reported a mean concentration of 1,726 ng/note for ketamine [17]. Previous studies have shown that ketamine is used as an adulterant in cocaine and methamphetamine [30, 31].

**Fig. 3:**
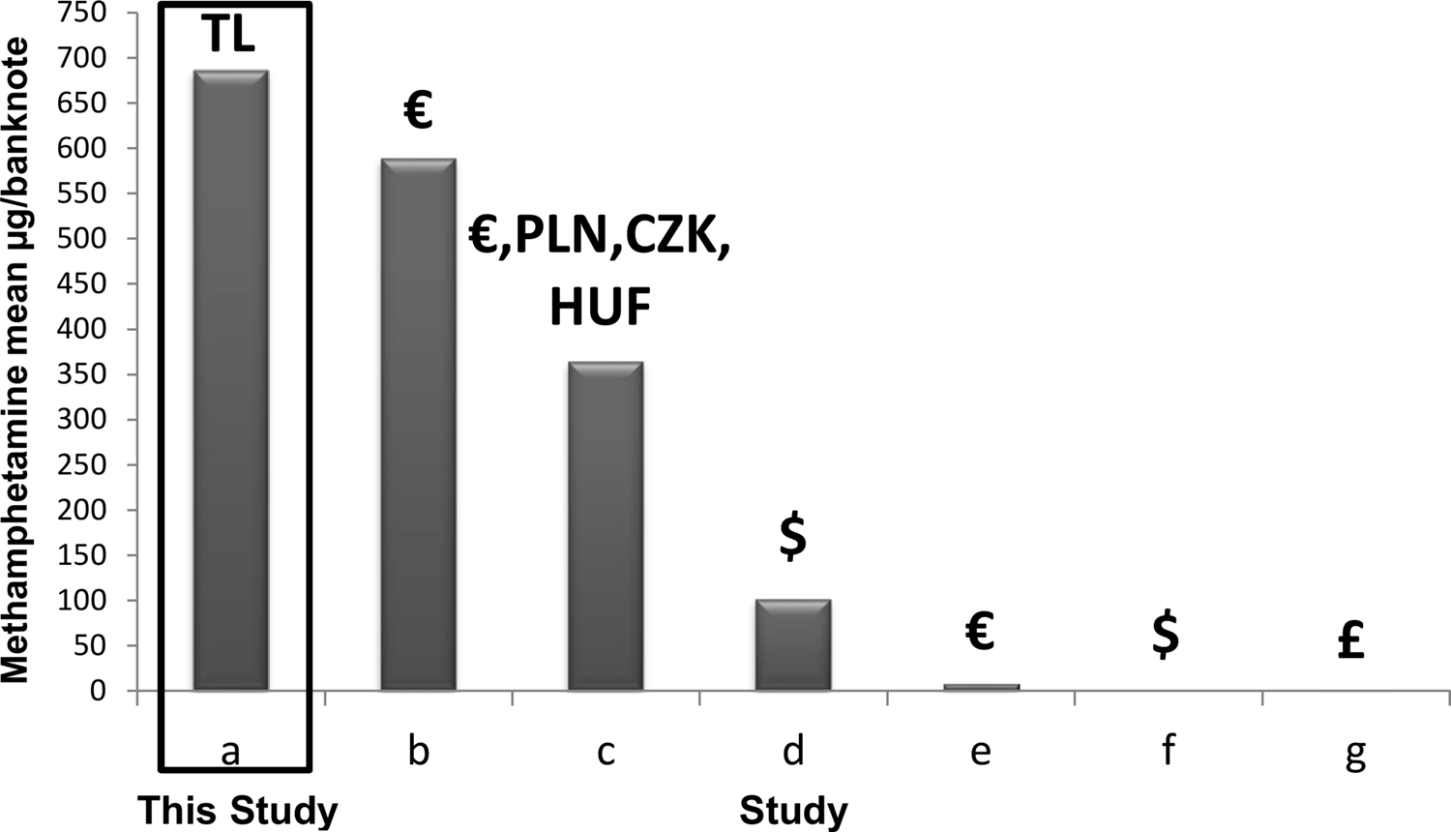
Reported methamphetamine (µg/banknote) mean concentrations on banknotes. **a** The present study 2023; **b** (16); **c** (16); **d** (17); **e** (4); **f** (27); **g** (6)

Methamphetamine is the most widely used and distributed synthetic drug globally, with its production and consumption steadily increasing in Southeast Asia, North America, Southwest Asia, Africa, and Europe [2, 28, 31]. Depending on its form, methamphetamine can be taken orally, injected, or smoked using pipes. In Turkey, the prevalence of crystalline forms, commonly known as crystal meth, and the consumption of methamphetamine are exceptionally high [32]. Figure 3 illustrates the concentration of methamphetamine across all examined banknotes. The high levels of methamphetamine contamination in the banknotes underscore the widespread use of this substance in Turkey. Methamphetamine was detected in all banknotes, with a notably broad detection range of 6.9–10,644 ng/note and a mean concentration of 660.7 ng/note in Turkey.

In Europe, Mackul'ak et al. conducted a comparative study on banknotes used in the Czech Republic and Slovakia, reporting significantly higher concentrations of methamphetamine at levels 940 and 760 ng/note, respectively [16]. Additionally, Ieong et al. reported the presence of methamphetamine at a mean concentration of 100.8 ng/note [25]. Wimmer and Schneider [-[4], Luzardo et al. [27], and Bowdler et al. [6] reported low levels of methamphetamine, with a mean value of 7 ng/note, 0.91 ng/note, and 0.779 ng/note, respectively, as depicted in Fig. 3. In the present study, the level of methamphetamine contamination was notably high, and the average concentration closely resembled that of Slovakian banknotes reported by Mackul'ak et al. [16]. Mackulak et al. suggested that banknote contamination levels and substance types align with drug abuse trends [16], a finding consistent with the results of the present study and previous investigations into drug abuse trends in Turkey [32].

Amphetamine was detected on 18.3% of all banknotes, with a detection range of 1.5–840.5 ng/note and a mean concentration of 46.0 ng/note. Luzardo et al. reported relatively low amphetamine levels, with a mean concentration of 1.4 ng/note [27]. MDMA was found in 21.2% of all banknotes, exhibiting a detection range of 25.2–696.6 ng/note and a mean concentration of 65.9 ng/note. Bowdler et al. [6] and Wimmer and Schneider [4] reported mean concentrations of MDMA at 62.8 µg/banknote and 9.0 ng/note, respectively. The degradation product of heroin, 6-AM, was found in 21.2% of all samples, with a detection range of 10.5–1860.9 ng/note and a mean concentration of 120.6 ng/note. Wimmer and Schneider reported 6 AM with a mean concentration of 15.5 ng/note [4]. Morphine was found in 9.3% of all banknotes, displaying a detection range of 2.3–506.2 ng/note and a mean concentration of 22.9 ng/note. Wimmer and Schneider reported a mean morphine concentration of morphine at 16.5 ng/note [4]. Codeine was present on 4.8% of all banknotes, with a detection range of 1.8–34.9 ng/note and a mean concentration of 6.3 ng/note. Both morphine and codeine are related to heroin in the presence of 6-AM. THC was detected in 4.0% of all banknotes, with a detection range of 9.1–235 ng/note and a mean concentration of 107.4 ng/note. Heroin was not detected in any of the samples. Several illicit drugs, including THC, heroin and cocaine, can degrade in the presence of moisture and air, and the detection of degradation products in currency may be attributed to recent contact with those drugs [33].

The synthetic cannabinoid MDMB-4en-PINACA was detected on 45.3% of all banknotes, with a detection range of 0.5–9.0 ng/note and a mean concentration of 1.3 ng/note. The synthetic cannabinoid ADB-BUTINACA was detected in 4.6% of all banknotes, displaying a detection range of 0.5–6 ng/note and a mean concentration of 1.1 ng/note. Interestingly, no prior reports have mentioned the new psychoactive substances. Tramadol was detected in only three banknotes from Adana, with a detection range of 67.9–945.1 ng/note and a mean concentration of 595.4 ng/note. Pregabalin was detected in seven banknotes, with a detection range of 35.6–402.8 ng/note and an average concentration of 179.4 ng/note in Adana and Ankara, respectively.

## Discussion

Significant differences were identified between banknotes of lower denominations (5, 10, 20, and 50 TL) and higher denominations (100 and 200 TL) for amphetamine, methamphetamine, MDMA, cocaine, benzoylecgonine, and ketamine (p < 0.005). Drug contamination in high-denomination banknotes was significantly lower than that in low-denomination banknotes. No significant differences were observed among the banknotes of low denominations (5, 10, 20, and 50 TL). Generally, banknotes of low denominations exhibited higher drug concentrations, potentially explained by their increased circulation rates and/or extensive use in street deals. For 6-AM, morphine, and codeine, significant differences were observed among all denominations (p < 0.05). In the case of THC, MDMB-4en-PINACA, and ADB-BUTINACA, no significant differences were observed between the banknotes of different denominations. Pregabalin and tramadol were not tested due to small sample sizes.

The drugs, number of positive samples, and mean concentrations in the three cities are summarized in Table 2. The mean concentrations of ADB-BUTINACA, MDMB-4en-PINACA, THC, amphetamine, and MDMA in banknotes were similar across the three cities (*p* > 0.05). The mean concentrations of methamphetamine, cocaine, ketamine, and benzoylecgonine were comparable in Ankara and Adana but significantly higher in Istanbul (*p* < 0.001). Significant differences were also observed in the mean concentrations of morphine and 6-AM across the cities, with the highest concentrations observed in Ankara. The mean concentrations of codeine were similar in Istanbul and Adana, but those in Ankara were significantly higher than in other cities (*p* < 0.001).

**Table 2:** Positive sample counts, mean concentrations, and range of concentrations for each compound

Compound	Number of Positive Samples	% of Positive Samples	Mean (ng/banknote)	Minimum /Maximum
Ankara				
ADB-BUTINACA	8	4	1.2	0.5–4.8
MDMB-4en-PINACA	100	50	1.3	0.5–5.6
THC	8	4	105.3	11.6–191
Amphetamine	34	17	52.1	20.0–840.5
Methamphetamine	200	100	347.5	31.2–2106.9
MDMA	23	12	66.9	25.2–696.6
Cocaine	200	100	340.7	13.9–1972.6
Benzoylecgonine	200	100	156.3	3.2–1685.5
Ketamine	200	100	13.3	2.6–62.0
Tramadol		0		
6-AM	37	19	159.4	10.5–1860.9
Morphine	14	7	56.8	2.5–506.2
Codeine	8	4	15.9	4.1–34.9
Pregabalin	3	2	174.2	35.6–402.8
Heroin		0		
Adana				
ADB-BUTINACA	9	5	1.2	0.5–2.4
MDMB-4en-PINACA	61	31	1.1	0.5–3.9
THC	7	4	109.2	9.0–225.3
Amphetamine	35	18	46.2	20.1–207.1
Methamphetamine	200	100	375.6	54–2253.6
MDMA	48	24	71.1	26.2–243.5
Cocaine	200	100	335.5	4.3–7391.4
Benzoylecgonine	200	100	163.8	1.3–2049.7
Ketamine	200	100	13.6	1.7–48.1
Tramadol	3	2	595.4	67.9–945.1
6-AM	44	22	119.2	10.7–848.0
Morphine	20	10	14.6	2.3–38.3
Codeine	11	6	2.5	1.8–6.2
Pregabalin	4	2	183.3	39.7–369.7
Heroin		0		
Istanbul				
ADB-BUTINACA	11	6	1.1	0.5–6.0
MDMB-4en-PINACA	111	56	1.3	0.5–32.1
THC	9	5	107.8	29.0–235.0
Amphetamine	41	21	40.7	1.5–101.9
Methamphetamine	200	100	1258.9	6.9–10,644.0
MDMA	56	28	61	30.2–534.6
Cocaine	200	100	750.4	2.3–8438.0
Benzoylecgonine	200	100	228.6	1.0–3241.0
Ketamine	200	100	82.55	0.6–622.4
Tramadol				
6-AM	46	23	90.9	11.6–675.3
Morphine	22	11	8.8	2.6–29.1
Codeine	11	6	3	1.8–4.2
Pregabalin		0		
Heroin		0		

As shown in Table 2, cocaine, methamphetamine, ketamine, and benzoylecgonine were detected in all samples, indicating repeated direct contamination with drugs (methamphetamine, 10,644 ng/note; cocaine, 8438 ng/note). Previous studies indicated that mechanical currency counters in financial institutions, ATMs, storage of banknotes, and the transfer of banknotes in public activities contribute to contamination [28]. Trends in licit and illicit drug abuse during the COVID-19 pandemic in Turkey were reported in 2022 [40]. In this study, synthetic cannabinoids were the most commonly detected abused substances (47.8%), followed by THC (32.7%), methamphetamine (32.1%), heroin (9.3%), MDMA (7.9%), pregabalin (7.4%), and cocaine (6.2%). The trends of drug residues in this study align with those reported [32]. Although, THC was not a commonly contaminated residue, it was the second most commonly abused illicit drug. This can be explained by the fact that it is sold through joints, which prevents contamination. Due to changing black market distribution patterns and changes in supply, the names of synthetic cannabinoids have been changed and updated in this study. Extensive methamphetamine use could result in notable contamination of this substance on banknotes (Fig. 3). While recent studies have revealed significant changes in methamphetamine use compared to previous findings, the lack of current research prevents making conclusive determinations about the contamination with methamphetamine prevailing worldwide. Low levels of cocaine contamination on a banknote can be explained by Turkey's role as a transit route for cocaine rather than individual use of this substance (Fig. 2) [34, 35].

A total of 4411 cases were examined between 1 January 2022 and 31 December 2022 at the Narcotics Department of the Council of Forensic Medicine. A total of 74.4% (n = 3280) of the cases were positive for abused drugs. Cannabis was detected in 1142 cases (35.35% of all cases). In addition to cannabis, methamphetamine was detected in 1090 cases (27.5% of all cases), cocaine in 121 cases (3.1% of all cases), MDMA in 108 cases (2.7%), amphetamine in 64 cases (1.6% of all cases) and opium gum in 20 cases (0.5% of all cases). The drugs seized were consistent with those detected on the banknotes.

This study had some limitations. First, it was challenging to control for potential cross-contamination between different currencies stored within the same wallet. Second, the absence of prior data on Turkey hindered the possibility of conducting a comparison across different periods.

## Conclusion

This recent application of LC–MS/MS methodology has successfully enabled the identification and quantification of 14 drug residues on Turkish banknotes for the first time. Employing a straightforward, rapid, and non-destructive technique for banknote preparation, this study sheds light on the current landscape of drug contamination on Turkish banknotes in circulation across major cities, such as Adana, Istanbul, and Ankara. Notably, the prevalent contaminants on Turkish banknotes were methamphetamine and cocaine. The concentration of cocaine was lower, while that of methamphetamine on banknotes was significantly higher than in previous studies. In addition, methamphetamine contamination levels were similar in Adana and Ankara but were significantly higher in Istanbul. The examination of contaminated banknotes emerges as a valuable tool for evaluating the prevalence of drug abuse and detecting the presence of newly emerging drugs in a specific region. In this study, the analysis of synthetic cannabinoids ADB-BUTINACA and MDMB-4en-PINACA as emerging drugs revealed a surprisingly high prevalence of contamination on banknotes. Drug residues on banknotes can indicate the type and prevalence of substances used in a given population. The presence of these illicit drugs, either individually or in complex mixtures on banknotes, raises concerns about potential pharmacological activities, and exposure to these may have adverse effects on human health. Due to the toxicological effects of exposure, particularly through the skin and lungs, employees who handle banknotes directly should be screened.
